# Neoadjuvant chemotherapy in 13 patients with locally advanced poorly differentiated thyroid carcinoma based on Turin proposal - a single institution experience

**DOI:** 10.1515/raon-2015-0001

**Published:** 2015-08-21

**Authors:** Nikola Besic, Marta Dremelj, Andreja Schwartzbartl-Pevec, Barbara Gazic

**Affiliations:** 1 Department of Surgery; 2 Department of Radiotherapy; 3 Department of Nuclear Medicine; 4 Department of Pathology, Institute of Oncology Ljubljana, Ljubljana, Slovenia

**Keywords:** poorly differentiated thyroid carcinoma, neoadjuvant, chemotherapy, survival, pathology

## Abstract

**Background:**

There is a paradigm that chemotherapy is ineffective in thyroid carcinoma. The aim of our study was to find out whether neoadjuvant chemotherapy before thyroid surgery had an effect on the size of primary tumour in patients with poorly differentiated thyroid carcinoma (PDTC) based on Turin proposal.

**Patients and methods.:**

Altogether, 13 patients (8 women, 5 men; median age 61 years) with PDTC based on Turin proposal were treated with neoadjuvant chemotherapy between 1986 and 2005. Tumour diameter was from 4.5 to 18 cm (median 9 cm). Regional and distant metastases were detected in 6 and 9 patients, respectively. Eight patients had pT4 tumour.

**Results:**

Altogether, 29 (range 1–5) cycles of chemotherapy were given. Tumour diameter decreased in all the patients and by more than 30% in 5 patients (= 38%). Two of these five patients had also preoperative external beam irradiation (EBRT). Total thyroidectomy, lobectomy and neck dissection were performed in 10, 3 and 5 cases, respectively. R0 and R1 resection was done in 5 and 8 cases, respectively. Eight patients had postoperative EBRT of the neck and upper mediastinum. The 5-year and 10-year cause-specific survival rates of patients were 66% and 20%, respectively.

**Conclusions:**

After neoadjuvant chemotherapy a partial tumour regression was observed in 38% of patients with PDTC based on Turin proposal.

## Introduction

A clinicopathologic entity of poorly differentiated thyroid carcinoma (PDTC) was proposed by Sakamoto *et al*. 30 years ago.^1^ They found that the differences in the survival rates among well differentiated, poorly differentiated and anaplastic carcinomas were significant and of value in determining management and survival of thyroid cancer patients.^1^ The World Health Organization (WHO) classification of tumours of endocrine organs in 2004 introduced PDTC as a separate entity and defined it as a follicular-cell neoplasm showing limited evidence of structural follicular cell differentiation, and morphologically and behaviourally at an intermediate position between differentiated (follicular and papillary carcinomas) and undifferentiated (anaplastic) carcinoma.^2^ At an international consensus meeting, uniform diagnostic criteria for PDTC (Turin Proposal criteria) were defined in the presence of solid/trabecular/insular growth pattern, absence of conventional nuclear features of papillary carcinoma, and in the presence of at least one of the following features: convoluted nuclei, mitotic activity ≥3/10 high-power fields, or tumour necrosis.^3^

PDTC is a rare disease that carries a poor prognosis.^4^ The incidence of PDTC as defined by the Turin Proposal criteria in Japan, USA and Northern Italy was 0.3%, 1.8% and 6.7%, respectively.^5^,^6^ In the literature, there are only limited data on the treatment of patients with PDTC.^7^–^11^ Recently, Ibrahimpasic *et al*. reported the results of treatment of 27 patients with PDTC presenting with gross extrathyroidal extension during the period 1986–2009 at the Memorial Sloan-Kettering Cancer Center.^9^ The majority of their patients (60%) who presented with or subsequently developed distant metastases eventually died of distant disease, therefore they concluded that systemic therapies to target distant metastatic disease are required to achieve improvements in the outcome.^9^ The aim of the present study was to find out whether neoadjuvant chemotherapy before thyroid surgery had an effect on the size of primary tumour in patients with locally advanced and/or initially metastatic PDTC based on Turin proposal.

## Patients and methods

During the period 1979–2005, 45 patients (33 women, 12 men; mean age 61.6 years) were treated with neoadjuvant chemotherapy for thyroid carcinoma at our tertiary comprehensive cancer center.^12^,^13^ Among them, 13 patients (8 women, 5 men; median age 61 years) had poorly differentiated thyroid carcinoma. They were initially treated during the period 1986–2005. The histological slides of all 13 patients with PDTC, who were the subject of this study, were reviewed by a pathologist experienced in thyroid pathology. All tumours fulfilled the Turin proposal criteria.^3^

In all patients in the primary chemotherapy group, the tumour was considered inoperable because of infiltration into the surrounding tissues. Altogether, ten patients were treated with neoadjuvant chemotherapy, while two patients were treated with preoperative chemotherapy and preoperative external beam radiotherapy (EBRT). Surgery was performed whenever the tumour was reduced after chemotherapy and/or EBRT and the surgeon deemed it resectable. The median interval between the beginning of chemotherapy and the surgical procedure was 30 days (range 8–85 days).

Data on the patients’ gender, age, history and extent of the disease, morphological characteristics, therapy, locoregional control, and survival were collected. The tumour size, presence of regional or distant metastases and residual tumour after surgery were assessed by TNM clinical classification according to the Union for International Cancer Control (UICC) criteria from 2009.^14^ Clinical characteristics of patients with PDTC based on Turin proposal and therapy are presented in Table 1.

The aim of the study was to evaluate the effect of chemotherapy in patients with PDTC. Because of rarity of PDTC we collected data of patients who were treated at our Institute and were included in the following consecutive clinical trials: Rational diagnostic and therapy of patients with thyroid tumours (J3-7842), Importance of cytomorphology, measurements of DNA, Ki 67 and apoptosis for planning and evaluation of effect of chemotherapy in thyroid carcinoma (J3-3026), Genetic and radio-nuclear methods in diagnostics and therapy of thyroid carcinoma (J3-3460), all supported by the Ministry of Science of Slovenia. The Medical Ethics Committee of the Republic Slovenia and the Protocol Review Board and Ethics Committee of the Institute of Oncology Ljubljana reviewed and approved all three studies, which were performed in accordance with the ethical standards laid down in an appropriate version of the 1964 Declaration of Helsinki. These studies were conducted with the understanding and consent of the involved human subjects. Our chart review for the present publication was approved by the Institutional Review Board.

### Surgery

Surgery was considered the most effective treatment of PDTC and has therefore remained its mainstay. At our Institute, treatment of each patient with advanced thyroid carcinoma depends on the decision of the multidisciplinary team consisting of a surgical oncologist, a specialist in nuclear medicine, a medical oncologist and a radiotherapist. Our patients were treated with surgery, radioiodine (RAI), EBRT, chemotherapy, or a combination of these modalities as dictated by the circumstances.^12^ Before RAI ablation of thyroid remnant and RAI therapy all patients were on low-iodine diet for two weeks in order to achieve moderate iodine deficiency.^15^

### Chemotherapy and radiotherapy

The treatment was started with a non-toxic schedule (*i.e.* 2 mg vinblastine over 12- or 24-h infusions in 1000 mL of 0.9% saline) as already reported in our previous publications.^8^,^12^,^13^,^16^ Vinblastine shows a cytostatic effect in cell lines models, which is reflected in rapid decrease of relative cell viability during prolonged exposure.^17^ At all tested concentrations, the relative cell viability was reduced by 20% or more already after 48 h exposure.^17^ However, vinblastine may cause cardiac arrhythmia, therefore we did not use vinblastine in a patient (number 7) with ischemic heart disease. Instead, in this patient, 20 mg of adriamycin in a 2-hour infusion was used once a week. In such doses, adriamycin does not cause nausea, vomiting, alopecia, hematopoietic side effects or congestive heart failure according to our extensive experience with adriamycin in patients with anaplastic thyroid carcinoma. The tumour increased in one of our patients (number 8) despite treatment with vinblastine, therefore a combination of vinblastine and cisplatin of 90 mg over a 24-h infusion with EBRT was used. After treatment with this combination, the tumour size decreased by more than half.

Hematologic (anaemia, leukopenia, neutropenia, and thrombocytopenia) and non-hematologic (nephrotoxicity defined by serum creatinine level, alopecia, and nausea/vomiting) toxic effects were evaluated according to the National Cancer Institute - Common Toxicity Criteria, version 4.0.

The local effect of chemotherapy used to be assessed by clinical findings only. The size of the primary tumour was measured clinically each day during the first week after chemotherapy and once a week thereafter during the visits to the outpatient clinic and before the next cycle of chemotherapy. Resectability of a tumour was clinically evaluated by a surgeon once a week. The extent of the disease and the potential effectiveness of chemotherapy were evaluated before the first chemotherapy and before the surgical procedure by clinical examination, X-ray, CT scan, ultrasonography and/or serum thyroglobulin (Tg) concentration measurements, as dictated by the circumstances. The overall effect of chemotherapy on the primary tumour size was defined according to Response Evaluation Criteria in Solid Tumours (RECIST) criteria^18^: (1) progressive disease (PD): at least a 20% increase in the sum of longest diameter of target lesions, taking as reference the smallest sum of longest diameter recorded before the treatment started; (2) stable disease (SD): neither sufficient shrinkage to qualify for partial regression nor sufficient increase to qualify for progressive disease; (3) partial response (PR): at least a 30% decrease in the sum of the longest diameter of target lesions; and as (4) complete response if the tumour disappeared.

According to our study protocol, if primary tumour progressed after chemotherapy, the patient was treated with a combination of EBRT and chemotherapy. Two patients received preoperative EBRT with a median dose of 35 Gy (range 30–40 Gy) over three to four weeks. In one patient, intubation was necessary one week after the initiation of external irradiation with a daily dose of 2.5 Gy. Altogether, eight patients had preoperative and/or postoperative EBRT of the neck and superior mediastinum with a total tumour dose of 30.6–59.4 Gy (median 50 Gy). The radiation field included the whole neck up to the level of the mastoid process, bilateral supraclavicular and infraclavicular regions, and the superior mediastinum using a ^60^Co unit and two opposed fields.

### Follow-up and survival

For all patients, follow-up examinations were performed at our Institute at least once a year. They consisted of obtaining a detailed medical history, a physical exam, and determination of the serum Tg concentration. Whenever the Tg concentration was elevated, imaging (X-ray, ultrasound, RAI scintigraphy, computed tomography, magnetic resonance imaging, bone scintigraphy and/or positron emission tomography - computed tomography (PET-CT) scan) was performed to determine the location and extent of residual disease or suspected recurrence.

Disease-specific survival was defined as the period from the first day of treatment with preoperative chemotherapy to the death or last follow-up of the patient. Overall survival was defined as the period from the first day of primary treatment preoperative chemotherapy to death of any cause or the last follow-up. Disease-free interval was defined as the period from the beginning of chemotherapy to recurrence or the last follow-up. The disease-free interval excludes those patients with distant metastases at initial presentation.

### Statistical analysis

Characteristics of patients and treatments were compared by Fisher’s exact or chi-square test, where appropriate. The age of the patients and size of their tumours were compared using the Mann-Whitney rank-sum test. Survival and disease-free intervals were compared using a log-rank test. Survival curves were calculated according to the Kaplan-Meier method. For statistical analysis, SPSS 16.0 for Windows was used.

## Results

### Patients

Tumour diameter was from 4.5 to 18 cm (median 9 cm). Regional and distant metastases were detected in 6 and 9 patients, respectively. Six patients had lung metastases and three patients had bone metastases. Eight (61%) patients had pT4 tumour (Table 2).

### Actual chemotherapy and toxicity

Chemotherapy consisted of vinblastine, vinblastine with adriamycin or vinblastine with cisplatin in 11, 1 and 1 cases, respectively. The interval between the first chemotherapy and surgical procedure was 1–12 weeks (median 4 weeks). Altogether, 29 (range 1–5) cycles of chemotherapy were given.

The following toxic effects of cisplatin were observed in our patient number 8: leukopenia grade 1, nausea/vomiting grade 1–2, nephrotoxicity grade 1 and alopecia grade 1. None of other patients had any toxic side effects of chemotherapy because doses of chemotherapy used were very low.

### Response to treatment, survival, additional treatment and follow up

**FIGURE 1.**
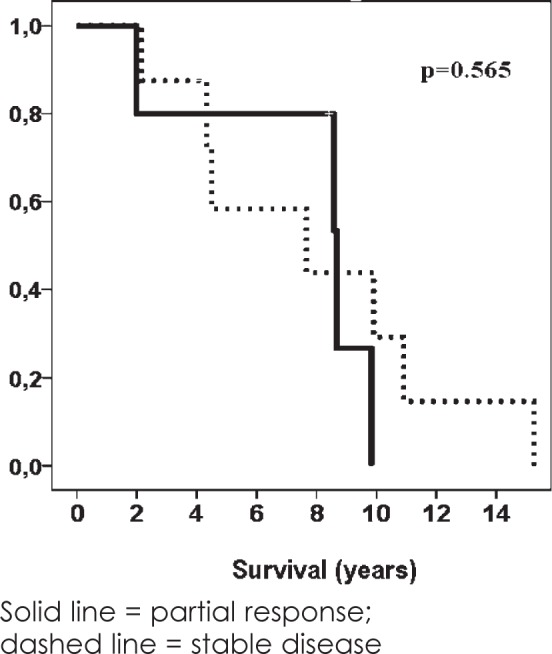
Survival of patients with poorly differentiated thyroid carcinoma (PDTC) according to the effect of chemotherapy.

Survival of patients with PDTC according to the effect of chemotherapy is presented in Figure 1. Tumour size decreased in all of patients, but PR was observed in 5 patients (38%). Two of these five patients had also preoperative EBRT.

Total thyroidectomy, lobectomy and neck dissection were performed in 10, 3 and 5 cases, respectively. R0 and R1 resection was done in 5 and 8 cases, respectively.

Radioiodine (RAI) therapy was used in patients with initially distant metastatic disease and distant dissemination during follow-up in 7 out of 9 and 3 out of 3 patients, respectively. They received 1–7 (median 2.5) therapies with RAI in a dose of 3.7–7.4 GBq. Eight patients received postoperative EBRT of the neck and upper mediastinum.

Distant metastases were diagnosed in three patients during follow-up of 7–189 months (median 118 months).

**FIGURE 2.**
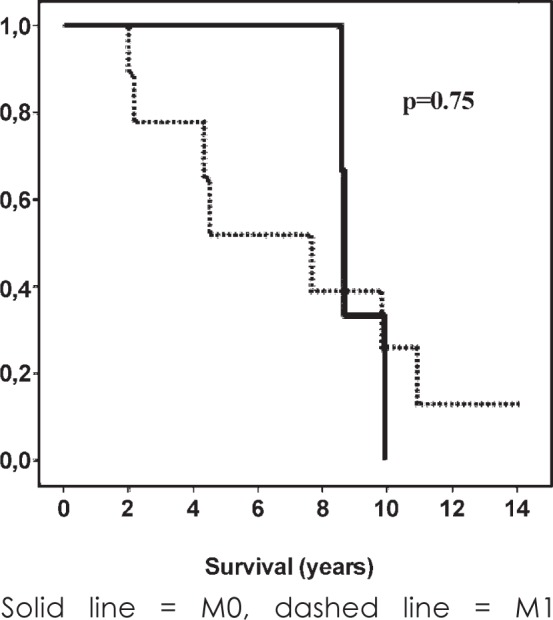
Survival of patients with poorly differentiated thyroid carcinoma (PDTC) and presence of metastases.

Ten patients died of distant metastases, one of distant metastases with small locoregional recurrence, and two patients died of other causes. The 5-year and 10-year cause-specific survival rates of patients were 66% and 20%, respectively. Survival of patients with PDTC based on Turin proposal and presence of metastases are shown in Figure 2.

## Discussion

Fortunately, aggressive locally advanced differentiated, poorly differentiated and anaplastic thyroid carcinomas are rare. However, because of this rarity, it is very unlikely that randomized trials will be conducted in patients with these rare tumours. One way to test the effectiveness of the therapy is to use a specific drug in a neoadjuvant setting. In two of our previous studies, we found out that neoadjuvant chemotherapy reduced the size of primary tumour by more than half in 44% of patients with differentiated thyroid carcinoma.^12^,^13^

The aim of the present study was to report the effectiveness of neoadjuvant chemotherapy in reducing the size of primary tumour in patients with locally advanced and/or initially metastatic PDTC based on Turin proposal. We found that in 38% of patients with PDTC based on Turin proposal, neoadjuvant chemotherapy decreased the size of primary tumour and PR was achieved. Based on this data we believe that chemotherapy may be the treatment of choice in locoregionally advanced and metastatic PDTC. Our data are not the only ones that oppose the paradigm that chemotherapy is ineffective in well, moderately or poorly differentiated thyroid carcinoma. Santini *et al*. reported a 37% response rate after a combination of carboplatin, epirubicin and thyroid-stimulating hormone (TSH) stimulation in fourteen patients with PDTC and RAI-resistant diffuse lung metastases.^19^ Carboplatin (300 mg/m^2^) and epirubicin (75 mg/m^2^) were given at 4- to 6-week intervals for a total of six courses. To raise serum TSH, either endogenous TSH stimulation was obtained by reducing the daily dose of L-thyroxin therapy or exogenous TSH stimulation was induced by recombinant human TSH. Lung computed tomography scans before and after therapy showed that one patient had a complete remission, while five patients had a partial remission.^19^

We believe that extensive surgery is not enough to obtain long-lasting locoregional control of disease in advanced PDTC. Namely, Ibrahimpasic *et al*. reported that 19 patients had only microscopic residual disease and 8 (42%) of them had locoregional recurrence.^9^ They also reported that 63% of patients had only RAI therapy, 11% underwent RAI therapy and EBRT, while 11% had only EBRT.^9^ On the other hand, our 8 patients with microscopic residual disease after thyroid surgery received a more extensive additional therapy: all of them had initial chemotherapy, postoperative RAI ablation of thyroid remnant and EBRT. Additionally, 5 patients (62.5%) also received RAI therapy. A more extensive additional therapy in our patients might be the reason that locoregional recurrence occurred in only 25% of cases.

Locoregional recurrence of thyroid carcinoma may lead to an uncontrollable disease and frequently often correlates with poor outcome. EBRT is used to prevent locoregional recurrence. According to the American Thyroid Association guidelines^20^, EBRT treatment of the primary tumour should be considered in patients aged over 45 years with grossly visible extrathyroidal extension at the time of surgery and a high likelihood of microscopic residual disease, or in patients with gross residual tumour in whom further surgery or RAI would likely be ineffective. In a recent review article, Sanders *et al*. concluded that EBRT should probably be considered also in patients with PDTC who have pT3 carcinoma, extracapsular extension of lymph node disease or extensive lymph node involvement.^7^

One reason that supports the initial multimodal approach is the fact that PDTC is often composed of a poorly differentiated as well as moderately differentiated component. It is well known that poorly differentiated cells are sensitive to chemotherapy. Neoadjuvant chemotherapy proved to be effective in all our patients, while in 38% PR of primary tumour was observed. RAI was used whenever possible to treat the differentiated component of PDTC.

Our study has several limitations. It is not randomized and there is no control group of patients (without chemotherapy). Furthermore, number of patients is too small to draw any conclusions whether the prognosis of R0 tumours is superior to that of R1 tumours and whether R1 tumours can be controlled by EBRT. However, we believe that in order to prevent locoregional recurrence and/or dissemination, the initial treatment should be based on prognostic and predictive factors also in patients with thyroid carcinoma. This principle is widely applied to many solid malignancies, *i.e*. breast cancer, colorectal cancer, head and neck cancer and many others. For example, after surgical procedure, a patient with breast cancer will also be treated with EBRT, chemotherapy, hormonal therapy and targeted therapy based on prognostic and predictive factors.^21^

However, American Thyroid Association and European Thyroid Association guidelines for treatment of differentiated thyroid carcinoma do not recommend initial multimodal approach in more aggressive types of differentiated thyroid carcinoma.^21^,^22^ It is well known that PDTC based on Turin proposal and anaplastic thyroid carcinoma are aggressive tumours that cause locoregional recurrence and dissemination^4^, therefore, we believe that an adequate initial multimodal treatment is justified. Naturally, in locoregionally advanced and/or metastatic PDTC, multimodal treatment should be used whenever possible. With such an approach, excellent locoregional control of disease was achieved in our patients. None of our patients had uncontrollable locoregional PDTC.

In locally advanced patients with PDTC based on Turin proposal from the Memorial Sloan-Kettering Cancer Center^9^, 5-year disease-specific survival was only 49%, while it was 66% in our patients, although initially distant metastases were more common in our series (37% *vs.* 61%). Possibly, longer survival of our patients was a result of our multimodal treatment approach. All our patients had initial chemotherapy, and 92% of them received adjuvant therapy: 31% RAI only, 15% EBRT only, while as many as 46% received both RAI therapy and EBRT. On the other hand, only 77% of patients reported by Ibrahimpasic *et al*. underwent adjuvant therapy: 55% RAI only, 11% EBRT only, while only 11% underwent both RAI therapy and EBRT.^9^

Jung *et al*. reported treatment results in 49 patients with PDTC not based only on Turin proposal.^23^ RAI therapy was used in 38 patients. Patients with RAI therapy had significantly longer survival in comparison to patients without RAI therapy (5-year survival: 73% vs. 60%).^22^ However, in a multivariate analysis, RAI therapy was not an independent factor for survival.^22^ Similarly, Lai *et al*. reported that RAI therapy was not an independent prognostic factor for survival in a retrospective review consisting of 82 patients with insular carcinoma, possibly because patients with worse-prognosis tumours were selected for a more extensive adjuvant treatment, obscuring any potential benefit.^10^ RAI scanning was performed at the Memorial Sloan-Kettering Cancer Center in eight patients with PDTC based on Turin proposal with distant metastases at presentation, and seven (87.5%) patients had RAI-avid metastases.^9^ Similarly, at our institute, 83% of cases with distant disease had RAI-avid metastases, so these patients underwent RAI therapy.

Like many others studies^7^,^9^,^10^,^23^ ours also shows that distant disease is the main cause of death in patients with locally advanced and metastatic PDTC. Initially or after disease progression, PDTC was a systemic disease in 92% of our patients. In order to treat systemic PDTC based on Turin proposal effectively, not only RAI but also other systemic treatment modalities are needed. Of course, there is a place for targeted therapy in PDTC, but at present time, there are only limited data about its use in poorly differentiated thyroid carcinoma.^24^ Sorafenib was reported to be effective treatment in radioiodine-refractory PDTC.^25^

## Conclusions

After neoadjuvant chemotherapy and preoperative EBRT a partial response of primary tumour was observed in 38% of patients with PDTC based on Turin proposal. Surgical procedure is the mainstay of treatment in PDTC, but postoperative RAI therapy, EBRT, or both, seem to be associated with prolonged survival.

## Figures and Tables

**TABLE 1.** Clinical characteristics and therapy of patients with poorly differentiated thyroid carcinoma (PDTC) based on Turin proposal

**Patient no.**	**Gender**	**Age**	**Histology of primary PDTC**	**TNM**	**Effect of chemotherapy (RECIST)**	**Preoperative (neck)**	**Surgical treatment**	**Residual tumour after surgery**	**RAI ablation of thyroid remnant**	**RAI therapy (number)**	**EBRT (neck)**	**Disease specific survival (months)**	**Cause of death**

						**EBRT**							
1	F	62	Hurthle cell	T3N1M1	SD	no	TT+mRND	R1	yes	0	no	131	DM
2	F	80	Papillary	T3N1M1	PR	no	TT+mRND	R1	yes	2	yes	24	DM
3	F	78	Papillary	T3N1M1	SD	no	TT+mRND	R1	yes	0	yes	26	DM+LR
4	F	45	Follicular	T4N0M0	SD	no	TT	R1	yes	1	yes	119	DM
5	F	47	Hurthle cell	T4N0M1	SD	no	TT	R1	yes	5	no	52	DM
6	M	69	Follicular	T4N1M0	PR	yes	TT	R1	yes	0	yes	101	Other causes
7	M	65	Papillary	T4N1M1	SD	no	lobectomy+mRND	R1	yes	2	yes	49	Other causes
8	M	61	Follicular	T4N0M0	PR	no	lobectomy	R0	yes	3	yes	104	DM
9	M	56	Papillary	T4N1M1	SD	no	lobectomy+mRND	R1	yes	6	yes	92	DM
10	F	57	Papillary	T4N0M0	PR	yes	TT	R0	yes	2	yes	103	DM
11	F	63	Follicular	T3N0M1	SD	no	TT	R0	yes	3	no	183	DM
12	F	37	Follicular	T3N0M1	SD	no	TT	R0	yes	1	no	54	DM
13	M	59	Follicular	T4N0M1	PR	no	TT	R0	yes	7	no	118	DM

DM = distant metastases; F = female; LR = locoregional disease; M = male; mRND = modified radical neck dissection; PR = partial response; RAI = radioiodine; SD = stable disease; TT = total thyroidectomy

**TABLE 2.** Presence of distant metastases in 13 patients with poorly differentiated thyroid carcinoma (PDTC) based on Turin proposal treated with neoadjuvant chemotherapy

**Factor**	**Subgroup**	**Without metastases (N = 4)**	**With metastases (N = 9)**	**p-value**
**Mean age (years)**		59	62	0.64
**Mean tumour size (cm)**		10	9.7	0.64
**Gender**	Female	2	6	1.00
	Male	2	3	
**Age (years)**	44 or less	0	1	1.00
	45 or more	4	8	
**Tumour diameter (cm)**	0 ≤ 4	0	0	1.00
	More than 4	4	8	
**pT tumour stage**	pT3	0	5	0.105
	pT4	4	4	
**N stage**	N0	3	4	0.56
	N1 or N2	1	5	
**M stage**	M0	4	0	-
	M1	0	9	
**Thyroid surgical procedure**	Total or near-total thyroidectomy	3	7	1.00
	Lobectomy	1	2	
**Residual tumour after surgery**	R0	2	3	
	R1	2	6	1.00
**Neck dissection**	No	4	4	0.105
	Yes	0	5	
**Radioiodine ablation after**	No	0	0	
**surgery**	Yes	4	9	1.00
**Therapy with radioiodine**	No	1	2	1.00
	Yes	3	7	
**Effect of chemotherapy**	< 50% or no effect	1	7	0.22
	50–99%	3	2	
**Recurrence**	No	1	0	
	Yes - distant	3	0	
	Disease present permanently	0	9	
**Outcome**	Death of disease	3	8	1.00
	Death of other causes	1	1	
**Disease-free interval in months: mean (range)**		106 (101–119)	-	-
**Cause-specific survival in months: mean (range)**		106 (101–119)	81 (24–183)	0.75
